# Evaluation of the In Vitro Wound-Healing Activity of Calabrian Honeys

**DOI:** 10.3390/antiox8020036

**Published:** 2019-02-06

**Authors:** Paolo Governa, Gabriele Carullo, Marco Biagi, Vittoria Rago, Francesca Aiello

**Affiliations:** 1 Department of Biotechnology, Chemistry and Pharmacy-Department of Excellence 2018-2022, University of Siena; Via Aldo Moro 2, 53100 Siena, Italy; paolo.governa@unisi.it; 2 Department of Pharmacy Health and Nutritional Sciences-Department of Excellence 2018-2022; University of Calabria; Edificio Polifunzionale, 87036 Arcavacata di Rende (CS), Italy; gabriele.carullo@unical.it (G.C.); vittoria.rago@unical.it (V.R.); 3 Department of Physical Sciences, Hearth and Environment; University of Siena; Via Laterina 8, 53100 Siena, Italy; biagi4@unisi.it

**Keywords:** wound healing, honey, diabetic foot ulcer, immunomodulation, HaCaT, Pinocembrin

## Abstract

The healing of skin wounds and particularly chronic wounds, such as diabetic foot ulcers, is still a clinical emergency. Despite the many therapeutic tools that are available so far, none seems to be really effective and safe. In this context, we highlighted the renewed wound healing activity of honey, a viscous aromatic and sweet food, by way of in vitro wound-healing assays, using the HaCaT cell line. Specifically, we investigated five monofloral or multifloral honeys from different Calabrian provinces using them as such or extracted (by Amberlite^®^ or *n*-hexane and ethyl acetate). The chemical composition of honeys was ascertained by ^1^H NMR spectroscopy and by the gas chromatography/mass spectrometry (GC/MS) method for volatile organic compounds (VOCs). Amongst the five tested honeys, BL1 and BL5 honeys showed the most promising healing properties. Pinocembrin, which was revealed in BL1 (multifloral) and BL5 (orange) honey samples, is a flavanol that is already known to possess interesting biological activities, including healing. This study aims to investigate how a traditional food such as honey, which is appreciated for its nutritional value and used in folk medicine, can be enhanced as an effective modern remedial to promote a multifaceted and safe healing activity for all skin wounds.

## 1. Introduction

The increase of diabetes incidence leads to a major development of one of its severe complications represented by diabetic foot ulcer [1]. It is well-known that the skin is our first barrier, and any lesion must be quickly healed to reduce the risk of infection. The skin of diabetes patients is different from those of healthy people, and shows a delayed healing process. During the wound-healing process, keratinocytes possess both regulatory and secretory functions, which are responsible for the re-epithelization of the skin. However, at a cellular level, the higher glucose exposure drives their morphological change, perhaps by a reduced sensitivity against growth factors, causing a dysregulation of the healing process [2]. The incomplete healing process leads to increasing infection development, higher inflammation state, reactive oxygen species (ROS) production, pain, and a change in the pH value of the wound. This scenario forced the use of antibiotic agents, which rapidly promote resistance [3]. Despite the development of several advanced wound care agents to promote the healing of chronic wounds, this is still a clinical emergency. In this context, the indigenous use of honey as wound-healing tool seems to be in line with an innovative and low-cost tendency, aiming to valorize the natural substances in severe health illness. Honey can be defined as a “galenic bee’s product”. This sweet animal compound is a concentrated of precious therapeutic components that are able to prevent and fight a plethora of human diseases. Indeed, honey has been reported to have an inhibitory effect on around 60 species of bacteria, as well as some species of fungi and viruses [4]. The antioxidant capacity of honey is important in many disease conditions, and is due to a wide range of compounds including phenolic compounds, peptides, organic acids, enzymes, and Maillard reaction products [4]. Honey has also been used in some gastrointestinal, cardiovascular, inflammatory, and neoplastic states. The amazing capability to change its composition from the different floral and local origin intrigues researchers worldwide to speculate regarding the new employment of honey. The human use of honey is traced to some 8000 years ago as depicted by Stone Age paintings. As a food, it is known by ancient years, and still nowadays is employed as a sweetener, but more interesting is its very versatile, safe, and low-cost curative aspect [5,6]. Noteworthy is the use of honey as a wound-healing agent, due to the biofilm-removing ability [7,8,9,10,11]. The debridement is a surgical painful procedure that is indispensable to remove biofilm before apply any healing medication [12,13]. Honey showed a facilitator effect during this intervention other than pain reduction. The mechanism responsible for this positive final effect include the ability to reduce inflammation and ROS production, to modulate the production of tumor necrosis factor-α (TNF-α), interleukin-1 (IL-1), and interleukin (IL-6) and mainly to maintain low pH values (around 3.5) [14]. Moreover, honey possesses antibacterial activity, and also stimulates keratinocytes proliferations and angiogenesis, promoting an efficacious skin-remodeling phase [15,16]. Among all of the different kinds of honeys, none before us have investigated the wound-healing properties of Calabrian honeys. We selected five kinds of honeys from different floral origins and provinces. They were analyzed by ^1^H-NMR spectroscopy and gas chromatography/mass spectrometry (GC/MS) analysis and assayed in an in vitro model of wound healing using the HaCaT cell line [17,18]. Also, as the immunomodulatory activity of honey has been shown to participate in its wound-healing effectiveness [19,20], we assayed the different Calabrian honeys for their effect in an in vitro corticosteroid-reduced keratinocytes proliferation model. The presence of pinocembrin in BL1 and BL5 honeys seems to be involved in their healing activities [21]. Pinocembrin was also isolated in a high amount from Glycirrhiza Glabra *var.* Cordara leaves and demonstrated its multi-target activities (antibacterial, antioxidant, and anti-proliferative) [22,23]. In this context, Pinocembrin was isolated from honey and assayed for its wound-healing activity, demonstrating a good biological profile.

## 2. Materials and Methods

### 2.1. Chemical Reagents

All the solvents, including diethyl ether, methanol, ethyl acetate, and *n*-hexane were purchased by Levanchimica srl (Bari, Italy). Amberlite® XAD-4, thin-layer chromatography (TLC, silica gel plates Merck 60 F254), and silica gel (0.040−0.063 and 0.063−0.200 mm) were purchased from Merck (Darmstadt, Germany).

### 2.2. Honey Types

**Figure 1:**
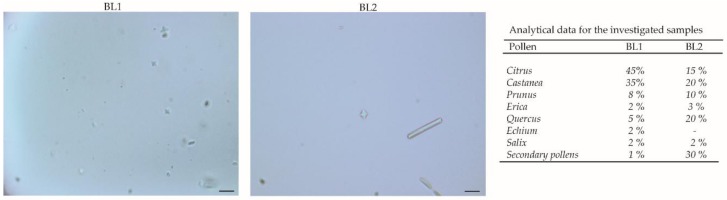
Melissopalynological analysis of multifloral honeys performed with an Olympus BX41 microscope; the images were taken with CSV1.14 software (RolandD Cyber Produksi, Hoogvliet Rotterdam, The Netherlands) using a CAM XC-30 for image acquisition. A minimum of 100 pollen grains were evaluated in each slide. Six slides were scored for each sample by two independent technicians.

Different types of Calabrian honeys were used: BL1H is a multifloral honey (acacia, lime, heather, chestnut, and citrus fruits) coming from Martirano (Catanzaro, Italy, 39°05′ N 16°15′ E), 350 m above sea-level (a.s.l.), 10 km from Tirrenian Coast; it was kindly donated by “Il Miele della Collina” of Giuseppe Pugliano. BL2H is a multifloral honey (wild fennel, acacia, thistle, rosemary, clover, citrus, plum, almond, cherry, and heather); BL3H is chestnut honey; while BL4H is acacia honey. BL2H, BL3H, and BL4H come from Taverna (Catanzaro, Italy, 39°01′ N 16°35′ E), 521 m a.s.l., 30 km from the Ionic coast. They were kindly donated by Vincenzo Badolato. BL5H is orange honey coming from Polistena (Reggio Calabria, Italy, 38°24′ N 16°04′ E), 254 m a.s.l., 20 km from the Ionic coast; it was kindly donated by “La boutique del miele” of Teresa Marcone. The multifloral origin was assessed by melissopalynological analysis [24] (Figure 1).

### 2.3. Extraction Procedure using Amberlite® XAD-4

According to published extraction methods [25], each honey sample (10 g) was diluted in 20 mL of acidified water (pH = 2 with HCl 37%) and filtered through cotton to remove solid particles. A column of five-cm diameter was loaded with Amberlite® XAD-4 (15 cm). Honey solution was passed through the column by elution with acid water (100 mL) and subsequently with distilled water (150 mL) to remove all of the sugars contained (monitoring by Molish assay). The phenolic components that remained in the column were then eluted with methanol (150 mL) and the methanolic solution dried under reduced pressure (40 °C). The residue was re-dissolved in 5 mL of water and extracted with diethyl ether (3 × 5mL). The combined organic layers were concentrated under reduced pressure and stored at −18 °C.

### 2.4. Liquid–Liquid Extraction

The selected BL1 and BL5 honeys were also extracted in a simple manner. Specifically, each honey sample (10 g) was diluted with distilled water (20 mL) and filtered through cotton to remove solid particles. Then, the solution was extracted with n-hexane (3 × 20 mL), and the *n*-hexane phase was concentrated under reduced pressure. The aqueous phase was further extracted with ethyl acetate (3 × 20 mL); the organic layer was concentrated under reduced pressure. Ethyl acetate and *n*-hexane extracts were stored at −18 °C.

### 2.5. Gas Chromatography/Mass Spectrometry (GC/MS) Analyses

Gas chromatography (GC) analyses were performed after dissolving the honey samples in methanol by using a Shimadzu GC17AGC equipped with a flame ionization detector (FID) controlled by Borwin Software (Shimadzu, Milan, Italy). Gas chromatography/mass spectrometry (GC/MS) analyses were performed by using a Hewlett-Packard 6890 gas chromatograph interfaced with a Hewlett-Packard 5973 Mass Selective, using electron impact (EI) (Agilent Technologies, Milan, Italy). The temperature of both the injector and detector was 250 °C. The initial oven temperature was set at 50 °C for three minutes. The temperature rate was set on 10 °C min^−1^ up to 250 °C.

### 2.6. ^1^H-Nuclear Magnetic Resonance Analysis (NMR)

^1^H-NMR spectra were recorded on a Bruker 300-MHz spectrometer with tetramethylsilane (TMS) as an internal standard: chemical shifts are expressed in δ values (ppm) and coupling constants (J) are expressed in hertz (Hz). The total honey samples BL1H and BL5H (5 mg) were diluted in 600 µL of deuterium oxide (D_2_O), while BL1E and BL5AE were diluted in 600 µL of chloroform-*d* (CDCl_3_). Spectra were obtained after 64 scans of 64 K data points at 20 °C using a spectral width of 13 ppm, an acquisition time of 2.0 s, recycle delay of 2.0 s, and a 90 °C flip angle.

### 2.7. Isolation of Pinocembrin

Pinocembrin was easily recovered from BL1NH through silica gel column chromatography using *n*-hexane/ethyl acetate (1/1) as the eluent. It was recovered under UV (254 nm) irradiation with TLC, compared with an in-house standard and characterized by GCMS and ^1^H,^13^C-NMR.

### 2.8. Cell Culture and Treatments

Human keratinocytes from adult skin (HaCaT) were cultured in 75 cm^2^ flasks (Sarstedt, Milan, Italy) in complete Dulbecco’s Modified Eagle’s medium (DMEM), which was composed of DMEM (Sigma-Aldrich) medium with 10% heat-inactivated fetal bovine serum (FBS, Sigma-Aldrich, Milan, Italy) and 1% L-glutamine (Sigma-Aldrich, Milan, Italy). EDTA-trypsin (Sigma-Aldrich, Milan, Italy) solution was used for detaching cells from flasks, and cell counting was performed using a hemocytometer by Trypan Blue staining. Samples were solubilized in dimethyl sulfoxide (DMSO) and diluted in DMEM in order to reach final concentrations of 10 mg/mL, one mg/mL, and 0.1 mg/mL for whole honeys and of 10 µg/mL, one µg/mL, and 0.1 µg/mL for honeys extracts. Pinocembrin was solubilized in DMSO and diluted in DMEM in order to reach a final concentration of 10 µM, one µM, and 0.1 µM.

### 2.9. Scratch Wound-Healing Assay

The scratch wound-healing assay was applied by slightly modifying the protocol of Chen (2012) [26], as previously described [27]. Briefly, HaCaT cells (5 × 10^4^) were seeded into six-well cell culture plates and allowed to grow to 70–80% confluence as a monolayer. The monolayer was gently scratched across the center of the well with a sterile one-mL pipette tip. A second scratch was performed in a perpendicular way to the first, creating a cross in each well. After scratching, the medium was removed, and the wells were washed twice in PBS (Sigma-Aldrich, Milan, Italy) solution. Fresh medium containing 5% V/V of heat-inactivated FBS and treatments was added to each well, and cells were grown for 24 hours. Images were obtained from the same fields immediately after scratching (t_0_) and after six hours and 24 hours using a Leica DMIL microscope and analyzed using ImageJ software by manually selecting the wound region and recording the total area.

The experiments were conducted in triplicate, and two fields were analyzed for each replicate (*n* = 6). Untreated scratched cells represented the control.

The percentage of wound closure was calculated using the following formula:

[(Wound area t_0_ − Wound area t)/Wound area t_0_] × 100

### 2.10. Cell Proliferation Assay

Cell proliferation assay was performed in basal condition by sulforhodamine B (SRB, Sigma-Aldrich, Milan, Italy) assay [28]. Briefly, HaCaT cells (1 × 10^4^) were seeded into 96-well plates and allowed to grow for 24 h. Cells were then treated with samples and incubated for six hours and 24 hours. Medium were then replaced, and cells were fixed by adding trichloroacetic acid to a final concentration of 10% (*v*/*v*) for one hour at 4 °C. Cells were washed, and 0.4% SRB solution was added to each well and incubated for 30 minutes at room temperature. Cells were then washed with 1% (*v*/*v*) acetic acid, and SRB was solubilized with 10 mM of Tris (Sigma-Aldrich, Milan, Italy, pH = 10.5). Absorbance was recorded at 540 nm using a MP96 spectrophotometer (Safas, Montecarlo). Treatments were performed in sextuplicate in three independent experiments, and cell proliferation was calculated by normalizing the absorbance of the test wells to the untreated control.

### 2.11. Cell Viability Assay

Cell viability assay was performed in a model of reduced cell proliferation induced by corticosteroid treatment by slightly modifying the protocol of Guichard et al. (2015) [29]. Briefly, HaCaT cells (1 × 10^4^) were seeded into 96-well plates and allowed to grow to 50% confluence. Cells were then treated with samples in the presence of 3 mM of 6α-methylprednisolone (MET) (Sigma-Aldrich, Milan, Italy) and incubated for six hours and 24 h. Medium were then removed, and cells were washed with phosphate buffered saline (PBS). Then, 100 µL of CCK-8 (Sigma-Aldrich, Milan, Italy) solution (1:10 in RPMI without phenol red) was added to each well and incubated for one hour at 37 °C. Absorbance was recorded at 450 nm using a MP96 spectrophotometer (Safas, Montecarlo). Treatments were performed in sextuplicate in three independent experiments, and cell viability was calculated by normalizing the absorbance of the test wells to the untreated control.

The absence of cytotoxic effect in MET-stimulated cells was assessed by measuring lactate dehydrogenase (LDH) release, as previously described [30].

### 2.12. Statistical Analysis

The statistical differences between the biological results were determined by one-way ANOVA test. Values are expressed in the range of +/− standard deviation and *p* < 0.05 was considered statistically significant. Graphs and calculations were performed using GraphPad Prism.

## 3. Results

### 3.1. Extraction

The five different honeys furnished the Amberlite® extracts (named BL1-5E) in good yields. In particular, starting from 10 g of honey samples, the yields were: BL1E (light orange oil, 63 mg), BL2E (yellow solid, 54 mg), BL3E (light brown solid, 42 mg), BL4E (white solid, 38 mg), BL5E (yellow oil, 59 mg). The honeys BL1 and BL5 were also extracted with *n*-hexane (NH) and ethyl acetate (EA). Starting from 10 g of BL1 and BL5, the yields were: BL1NH (white solid, 30 mg), BL1AE (orange solid, 32 mg), BL5NH (light yellow solid, 28 mg), and BL5AE (orange solid, 33 mg).

### 3.2. Analysis of Compounds

The samples presented a different composition in terms of organic acids, polyphenols, and other compounds. The different composition was verified by GC/MS and NMR analyses (Table 1 and Table 2). The substances that were found were amino acids and sugars, while the most interesting that was found with the two methods were pinocembrin and pinocembrin-7-methylether, which are present in BL1 and BL5 samples in good quantity. The fatty acids that were present in the samples were principally oleic acid (found in BL1NH, BL5NH, and BL5AE) and palmitic acid (found in BL1NH and BL5AE) [31,32,33].

The NMR spectra were recorded only for the total honeys (BL1H and BL5H) and for BL1E and BL5AE, because they demonstrated the most interesting biological profile.

### 3.3. Pinocembrin Data Analysis

**Figure 2:**
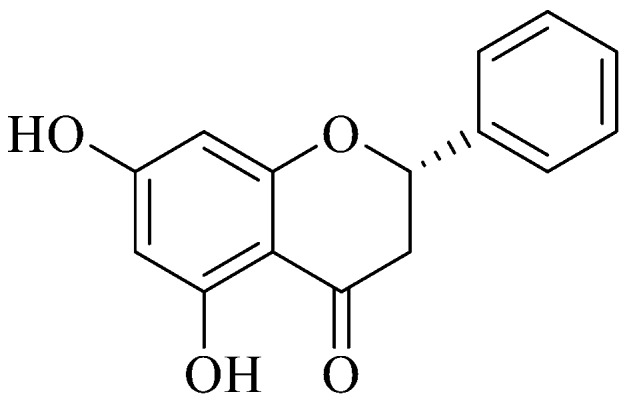
Steric configuration of (S)-pinocembrin or (S)-5,7-dihydroxy-2-phenylchroman-4-one.

Pinocembrin (Figure 2) was easily recovered from BL1NH through silica gel column chromatography. The data obtained were compared with those reported in literature. ^1^H NMR (CDCl_3_, 300 MHz): δ (ppm) 12.13 (b. s. 2H), 7.5–7.3 (m, 5H), 6.1 (s, 2H), 5.42 (dd, 1H, J = 3.0, 13.0 Hz), 3.10 (dd, 1H, J = 13.0, 17.2 Hz), 2.82 (dd, 1H, J = 3.0, 14.0 Hz). ^13^C NMR: δ (ppm) 195.7, 164.9, 164.3, 164.1, 138.2, 128.88, 128.86, 128.0, 126.13, 126.11, 103.0, 95.9, 95.5, 79.1, 43.2. GC/MS: 256 [M^+^]. Natural Pinocembrin is S-configuration with a specific rotation [α]D ⟨15⟩ of −45.3 (c, 0.9, acetone as solvent).

### 3.4. Scratch Wound-Healing Assay

In the attempt to dissect the wound healing capacity of honeys and their extracts, we applied an in vitro scratch wound-healing assay to the monolayer of HaCaT cells. The reliability of this in vitro method has been extensively demonstrated [34,35,36,37], even by comparison with in vivo experiments [38,39]. Moreover, the ability of untreated HaCaT cells to migrate toward the applied wound was confirmed in our model, and was enhanced by14% by TGF-β 4 ng/mL, which was used as the positive control according to literature data [40] (Table 3).

**Figure 3:**
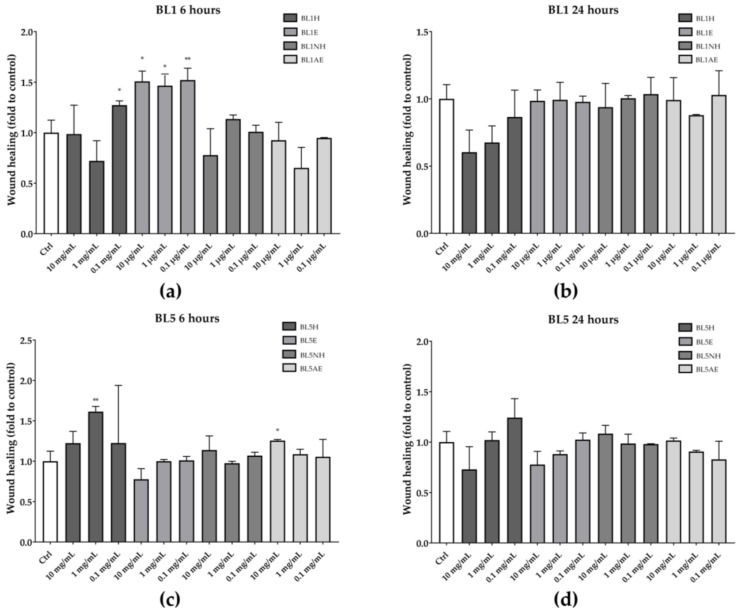
Wound-healing activity of BL1 (**a**, **b**) and BL5 (**c**, **d**) honeys and extracts after six hours (left) and 24 hours (right). * *p* < 0.05 vs. control; ** *p* < 0.01 vs. control.

Among the five tested honeys, only BL1 and BL5 were able to increase the wound healing rate after six hours of treatment, compared to the untreated control (Figure 3). In particular, BL1H at the lowest concentration used (0.1 mg/mL) and BL1E, at each of the tested concentrations, were able to increase the wound-healing rate by 27%, 51%, 47%, and 52%, respectively, compared to the untreated control. Instead, BL5H (1 mg/mL) and BL5AE (10 µg/mL) increased the wound-healing rate by 61% and 22%, respectively, compared to the untreated control. Neither BL1 nor BL5 were effective after 24 h of treatment.

**Figure 4:**
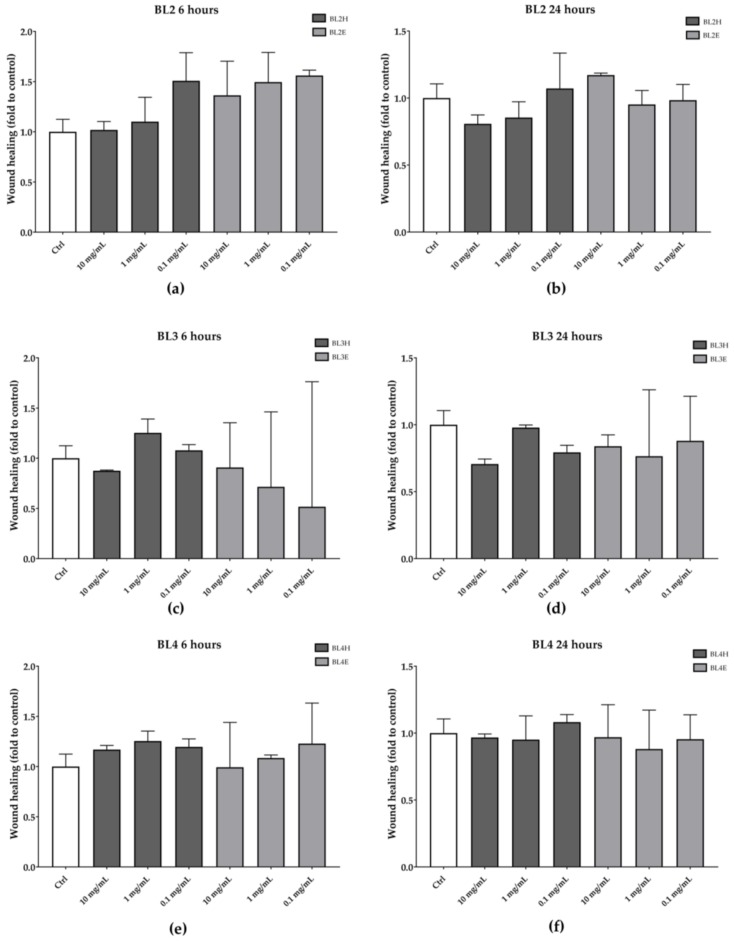
Wound-healing activity of BL2 (**a**, **b**), BL3 (**c**, **d**), and BL4 (**e**, **f**) honeys and extracts after six hours (left) and 24 h (right).

As reported in Figure 4, BL2, BL3, and BL4 were not effective in increasing the HaCaT wound healing at each time point.

**Figure 5:**
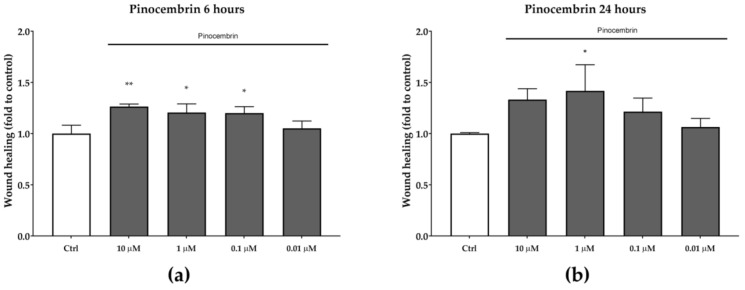
Wound-healing activity of pinocembrin after six hours (**a**) and 24 hours (**b**). * *p* < 0.05 vs. control; ** *p* < 0.01 vs. control.

Pinocembrin, which was identified in both BL1 and BL5 extracts, was also tested for its in vitro wound-healing activity. After six hours of treatment, pinocembrin was found to significantly increase the wound-healing rate by approximately 25% at each of the tested concentrations, compared to the untreated control (Figure 5). A similar trend was observed after 24 h, even if the statistical significance was obtained only at the concentration of one µM (*p* < 0.05).

**Figure 6:**
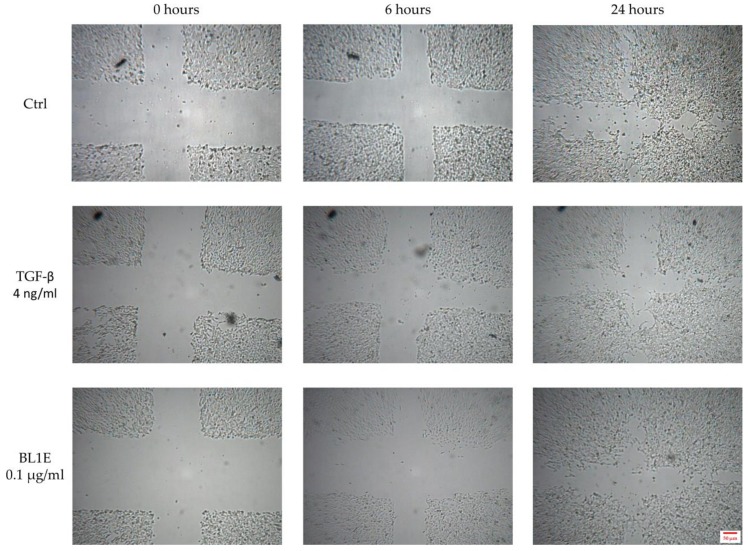
*In vitro* HaCaT wound healing of the untreated control (up), the positive control (center) and BL1E 0.1 µg/mL (down) at 0 (left), 6 (center) and 24 (right) hours after scratching.

Some representative pictures for the wound healing experiments are reported in Figure 6.

### 3.5. Cell Proliferation Assay

**Figure 7:**
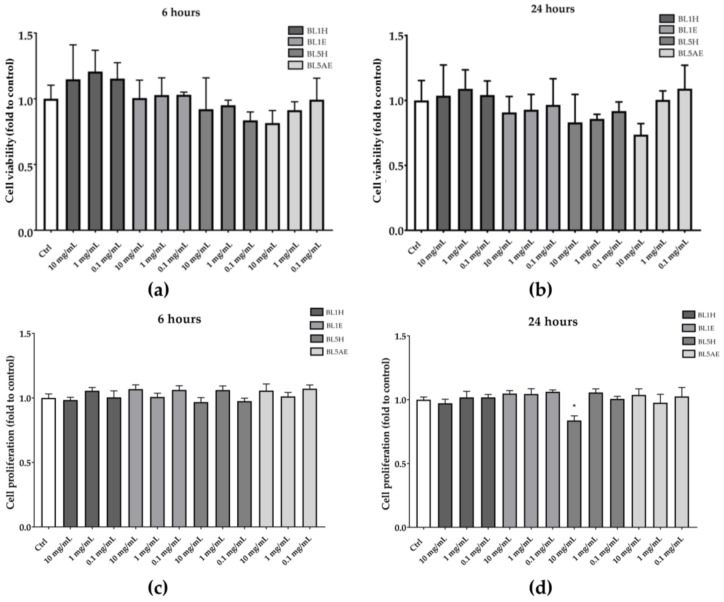
Effects of BL1H, BL1E, BL5H, and BL5AE on cell viability (**a**,**b**) and cell proliferation (**c**,**d**). * *p* < 0.05 vs. control.

Samples resulting in a significant wound-healing activity (i.e., BL1H, BL1E, BL5H, and BL5AE) were also tested for their ability to increase HaCaT cell proliferation. As reported in Figure 7, none of the tested samples was able to increase cell proliferation neither after six hours nor after 24 h, compared to the untreated control.

**Figure 8:**
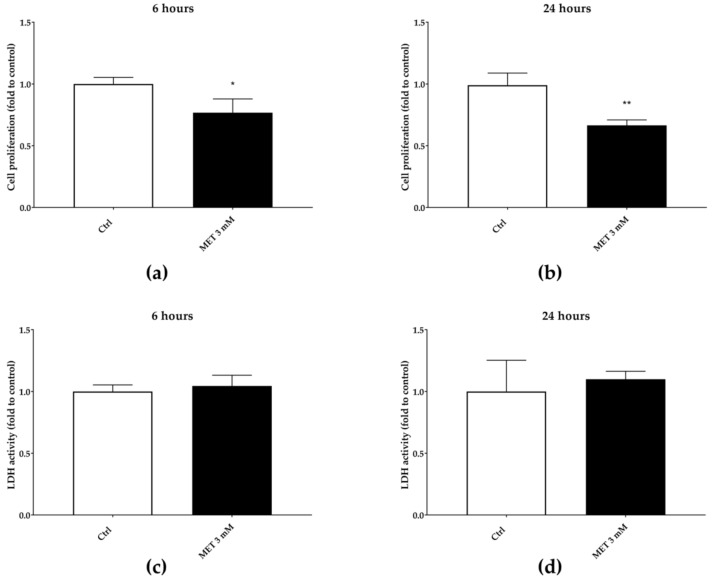
Three mM of 6α-methylprednisolone (MET) reduction of cell proliferation after six hours (**a**) and 24 h (**b**). The reduction of cell proliferation is not due to cytotoxic effect, as assessed by LDH extracellular release (**c**,**d**).

In order to evaluate whether the wound-healing effect is related to an immune-mediated mechanism, we tested the ability of BL1H, BL1E, BL5H, and BL5AE in a model of corticosteroid-reduced cell proliferation. Three mM of MET stimulation was able to significantly reduce cell proliferation at each time point, compared to the untreated control (Figure 8). However, this effect is not correlated with an increase in LDH activity in the supernatants, thus excluding cytotoxicity due to damages to the cell membrane.

**Figure 9:**
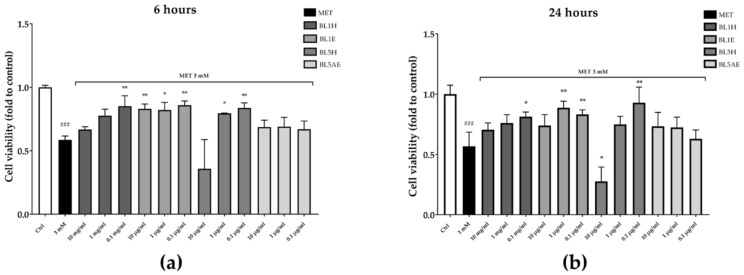
Protection from MET-impaired cell viability by BL1 and BL5 honeys and extracts after six hours (**a**) and 24 hours (**b**). * *p* < 0.05 vs. stimulus; ** *p* < 0.01 vs. stimulus; ^###^ *p* < 0.001 vs. control.

The reduction of cell proliferation was also confirmed by a reduction of cell viability observed by means of CCK-8 assay. As reported in Figure 9, after six hours, BL1H and BL1E reflected the results obtained in the wound-healing experiment, with BL1H (0.1 mg/mL) and BL1E (at each of the tested concentrations) reverting the MET-impaired cell viability by 45%, 42%, 40%, and 47%, respectively. BL5H was able to counteract the MET-impaired cell viability by 35% and 42% at the concentrations of one mg/mL and 0.1 mg/mL, respectively. After 24 h, BL1H (0.1 mg/mL) and BL1E (1 and 0.1 mg/mL) maintained their activity, whereas a BL1E 10 mg/mL protective effect was no more statistically significant. At 0.1 mg/mL, BL5H was still able to counteract the MET-impaired cell viability by approximately 64%.

**Figure 10:**
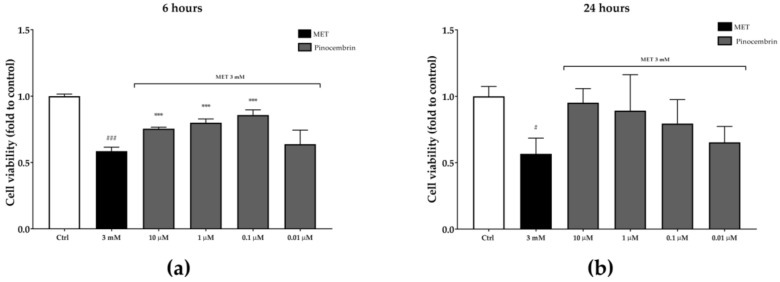
Protection from MET-impaired cell viability by pinocembrin after six hours (**a**) and 24 hours (**b**). *** *p* < 0.001 vs. stimulus; ^#^ *p* < 0.05 vs. control; ^###^ *p* < 0.001 vs. control.

Pinocembrin activity in the MET-impaired cell viability model was similar to that observed in the wound-healing assay. After six hours, pinocembrin counteracted the MET-impaired cell viability by 29%, 36%, and 46% at 10 µM, one µM, and 0.1 µM, respectively. Even though no statistical significance was obtained, pinocembrin activity was similar after 24 hours (Figure 10).

## 4. Discussion

In this work, the biological activity of different Calabrian honeys was tested in an in vitro wound-healing model using HaCaT cells. Among the five tested honeys, only two, namely BL1 and BL5, were able to induce significant keratinocytes migration and ameliorate wound healing. None of the tested sample was able to stimulate cell proliferation in basal condition, thus supporting a specific migration-inducing mechanism of action. Interestingly, the wound-healing activity was observed after a short exposure (i.e., six hours) and decreased after 24 h. This behavior is probably because wound healing in untreated cells was by far more evident after 24 hours, compared to six hours. Moreover, the wound-healing capacity of polyphenol-enriched products, such as honey, may result from an elaborate balance between the anti-inflammatory and immune-stimulatory effect of polyphenols [41]. At high concentration, or after prolonged exposure, polyphenols are able to reduce the production of pro-inflammatory cytokines and interact with cells metabolism and proliferation, thus reducing wound healing [27].

In the attempt to assess which classes of constituents may be related to the wound-healing activity of honey, we tested three different extracts obtained from both BL1 and BL5, one obtained by Amberlite® separation (BL1E and BL5E), and the other two by *n*-hexane (BL1NH and BL5NH) or ethyl acetate (BL1AE and BL5AE) extraction. Only BL1E and BL5AE were active, with BL1E being approximately twofold more effective in inducing HaCaT wound healing compared to BL5AE.

Pinocembrin was identified in both BL1 and BL5 extracts. Pinocembrin antimicrobial activity has been speculated as the main mechanism of action involved in its in vivo wound-healing effect [42]. Interestingly, in vitro pinocembrin was found to modulate the production of inflammatory cytokines, such as TNF-α, IL-1β, IL-6, and IL-10, by suppressing NF-κB and MAPK activation [43]. However, several other biological activities have been attributed to pinocembrin, both in vitro and in vivo, including a protective effect in stroke, Alzheimer’s, atherosclerosis, and cardiovascular diseases [44], together with anticancer and neuroprotective effects [45]. To the best of our knowledge, this is the first attempt to evaluate the wound-healing effect of pinocembrin in HaCaT cells. We found that pinocembrin may be able to accelerate in vitro skin wound healing by directly stimulating keratinocytes migration.

This positive activity may be useful to treat diabetic wounds, which are characterized by altered cytokines and growth factors levels and reduced keratinocytes and fibroblast migration and proliferation; all of these contribute to the lack of healing. The use of corticosteroids as anti-inflammatory drugs is a known practice to treat inflammation, but they are known to reduce cell proliferation by immunosuppressive mechanisms, thus reducing the wound-healing rate, especially in diabetic patients [46]. To simulate a difficult healing condition by reduced cell proliferation, we treated HaCaT cells with three mM of MET, and obtained a 35% decrease in cell proliferation, which corresponds to a 50% decrease in cell viability. Interestingly, both BL1H and BL5H were able to counteract the effect of MET on cell viability. Among the extracts, only BL1E was effective at each of the tested concentrations, and similar results were obtained with pinocembrin. Hence, the wound-healing effect of the BL1H, BL5H, and BL1E may be related to a modulation of the immune response, which would be consistent with data obtained with different honeys, as well as with other polyphenol-enriched natural products [47]. Moreover, this peculiar mechanism of action could be useful in difficult to heal wounds, such as diabetic wounds.

## 5. Conclusions

The evaluation of the in vitro wound healing activity of Calabrian honeys demonstrated a good performance especially for BL1E (multifloral honey from Tyrrenian coast), able to foster wound healing at each of the tested concentrations, compared to control. Furthermore, BL1H and BL1E were able to revert the MET-impaired cell viability, reflecting the results obtained in the wound-healing experiment. These interesting results could be attributed to the different pollen composition of BL1H, mainly represented by citrus and also to the presence of pinocembrin, which promoted wound healing *per se* in the same experimental conditions. Additional studies are ongoing to validate the mechanism of action involved in the wound healing activity, in order to validate BL1H and BL1E as new medical honey.

## Figures and Tables

**Table 1:** GC/MS analyses of honey extracts and percentage of volatile compounds (% in one mg of sample).

**Compounds**	**Identify Similarity**	**BL1E**	**BL1NH**	**BL1AE**	**BL5E**	**BL5NH**	**BL5AE**	**BL2E**	**BL3E**	**BL4E**
1-Methylisoquinoline	RI	0.32	-	0.18	-	-	-	-	-	-
1-Hexadecene	RI	-	-	-	-	-	-	0.37	-	-
1-Methoxy-4-propylbenzene	RI	0.75	2.33	-	-	0.63	-	-	-	-
1-(4-Aminophenyl-etyhanone	RI	-	-	-	-	-	-	-	1.62	-
2(1H)-quinolinone	RI	-	-	-	-	-	-	-	1.20	-
Ethyl benzoic acid	RI	0.50	-	-	-	-	-	-	-	-
Benzoic acid	RI/MS	6.61	-	0.79	11.98	-	-	-	5.36	7.94
4-Methoxy benzoic acid	RI	1.51	0.98	-	-	0.45	0.64	-	-	-
4-Methyl-1,1’-biphenyl	RI	0.31	-	-	0.38	-	-	-	-	3.34
2,4-Bis(1,1-dimethylethyl)-phenol	RI	0.82	-	-	-	-	-	-	-	1.72
2,5-Dipropyl-thyophene	RI	-	-	-	-	-	-	-	1.98	
4-Hydroxy-3,5,6-trimethyl-4-(3-oxo-1-butenyl)-2-cyclohexen-1-one	RI	0.50	2.32	-	-	-		-	-	-
Benzyl benzoate	RI	0.90	-	-	11.37	-	-	-	-	-
5-*Tert*buthyl-m-cymen	RI	-	-	-	-	-	-	-	-	29.1
6-Methoxy-6H-dibenzo[b,d]pyran	RI	1.25	1.90	0.18	0.36	0.08	1.80	-	-	-
6,7,8,9-Tetrahydro-2H-benzofuro(3,2-g)(1)benzopyrane-2-one	RI	-	-	-	-	-	-	-	1.80	-
Benzoic acid phenyl ester	RI	0.24	-	-	-	-	-	-	-	2.07
1-(4-Hydroxy-3,5-dimethoxyphenyl)-ethanone	RI	0.40	-	0.58	-	-	4.65	-	-	-
Eicosane	RI	-	-	-	-	-	-	0.25	-	-
Myristic acid	RI	-	-	-	-	-	-	2.80	-	-
Nonadecane	RI	-	-	-	-	-	-	1.30	-	-
Palmitic acid	RI/MS	-	0.50	-	-	-	0.13	-	-	-
Oleic acid	RI/MS	-	0.33	-	-	0.04	1.11	10.09	-	-
Octadecane	RI	-	-	9.27	-	-	-	-	-	-
1,1’-Methylenbis-benzene	RI	-	1.61	-	-	0.59	-	-	-	-
9-Eicosene	RI	-	0.78	-	-	-	-	-	-	-
4H-pyran-4one, 2,3-dihydro-3,5-dihydroxy-6methyl	RI	-	-	9.75	-	-	0.59	-	-	-
5-Hydroxymethyl-2-furancarboxaldehyde^*^	RI	-	-	42.3	-	-	-	-	-	-
5,5’-Oxy-dimethylene-bis(2-furaldehyde)	RI	-	-	6.32	-	-	-	-	-	-
Pentacosane	RI	-	-	8.10	-	-	-	-	-	-
Pinocembrin	RI/MS	0.63	9.09	1.08	1.13	-	0.98	-	-	-
Pinocembrin-7-methyleter	RI	0.32	-	2.99	0.74	-	1.95	-	-	-
Squalene	RI	-	-	3.64	1.54	-	-	-	-	-
Tritetracontane	RI	-	-	-	-	-	-	0.57	-	-
(Z)-9,17-octadecadienal	RI	-	-	-	-	-	-	0.39	-	-
(Z)-7-hexadecene	RI	-	-	-	-	-	-	0.47	-	-

RI, compounds identified on the basis of the retention index from the literature; MS, compounds identified by comparison of their mass spectra with the corresponding pure standards. *marker of higher temperature.

**Table 2:** ^1^H-NMR qualitative analyses of honey extracts.

Compounds	Multiplicity	δ H (ppm)	BL1H^a^	BL1E	BL5H	BL5AE
Proline (β, β’ CH_2_)	m	2.23	+	-	-	-
Proline (γCH2)	m	2.05	-	-	+	-
Succinic acid (α-β CH_2_)	m	2.68	+	-	-	-
Citric acid (CH_2_)	d	2.87	+	-	-	-
Malic acid (βCH_2_)	dd	3.06	+	-	-	-
β-Glucose (H4)	dd	3.40	+	-	+	-
Tyrosine (αCH)	m	3.43	-	+	-	-
α-Glucose (H4)	dd	3.45	-	-	+	-
Pinocembrin-7-methylether (OCH_3_)	s	3.90	-	+	-	+
β-Glucose (H1)	d	4.45	+	-	+	-
Fructose	m	4.00	+	-	+	-
β-Rhamonose (H1)	d	4.86	-	-	+	-
α-Glucose (H1)	d	5.02	+	-	+	-
Turanose (H1)	m	5.10	+	-	-	-
Maltose (H1)	m	5.30	+	-	-	-
Maltose (H1)	d	5.36	-	-	+	-
Sucrose (H1)	d	5.42	-	-	+	-
Chrysin (C6)	m	6.11	+	-	-	-
Tyrosine (H3, H5)	d	6.84	-	-	+	+
Phenyl acetic acid	m	7.10	-	-	-	+
Tyrosine (H2, H6)	s	7.20	-	-	+	+
Phenylalanine (Ph)	m	7.4–7.7	-	+	-	+
Pinocembrin (Ph)	m	7.40	-	+	-	+
Hydroxymethylfurfural (H3)	d	7.54	-	-	-	+
Pinocembrin (5OH)	s	12.00	-	-	-	+
γ-LACT-3-PKA (C8)	s	8.45	-	-	+	-

Footnote: detected compound (+), not detected compound (-).

**Table 3:** *In vitro* wound-healing rate of the untreated control (% of wound closure ± standard deviation). * <0.05 vs control.

Treatment	6 h	24 h
Ctrl	20.19 ± 2.53	52.36 ± 2.31
TGF-β 4 ng/mL	17.97 ± 0.44	59.84 ± 2.61 *
